# Visual Perception during Mirror-Gazing at One's Own Face in Patients with Depression

**DOI:** 10.1155/2014/946851

**Published:** 2014-11-20

**Authors:** Giovanni B. Caputo, Marco Bortolomasi, Roberta Ferrucci, Mario Giacopuzzi, Alberto Priori, Stefano Zago

**Affiliations:** ^1^DIPSUM, Università di Urbino, Via Saffi 15, 61029 Urbino, Italy; ^2^Unità Operativa di Psichiatria “Villa Santa Chiara”, Via Monte Recamao 7, Quinto di Valpantena, 37142 Verona, Italy; ^3^Fondazione IRCCS Ca' Granda-Ospedale Maggiore Policlinico, Dipartimento di Scienze Neurologiche, Università degli Studi di Milano, Via F. Sforza 35, 20122 Milano, Italy

## Abstract

In normal observers, gazing at one's own face in the mirror for a few minutes, at a low illumination level, produces the apparition of strange faces. Observers see distortions of their own faces, but they often see hallucinations like monsters, archetypical faces, faces of relatives and deceased, and animals. In this research, patients with depression were compared to healthy controls with respect to strange-face apparitions. The experiment was a 7-minute mirror-gazing test (MGT) under low illumination. When the MGT ended, the experimenter assessed patients and controls with a specifically designed questionnaire and interviewed them, asking them to describe strange-face apparitions. Apparitions of strange faces in the mirror were very reduced in depression patients compared to healthy controls. Depression patients compared to healthy controls showed shorter duration of apparitions; minor number of strange faces; lower self-evaluation rating of apparition strength; lower self-evaluation rating of provoked emotion. These decreases in depression may be produced by deficits of facial expression and facial recognition of emotions, which are involved in the relationship between the patient (or the patient's ego) and his face image (or the patient's bodily self) that is reflected in the mirror.

## 1. Introduction

Strange-face in the mirror illusions [1–4] are apparitional experiences that are produced by gazing at one's own face reflected in a mirror, under low illumination. In a study set-up, under controlled laboratory conditions, 50 healthy young adults, after about one minute of mirror-gazing, began to perceive strange-face apparitions [1]. These included huge deformations of one's own face (reported by 66% of individuals), a monstrous face (48%), an unknown person (28%), an archetypal face (28%), a face of a parent or relative (18%), and an animal face (18%).

Recently, Caputo et al. [5] showed that some schizophrenic patients perceived much more intense strange-face apparitions than healthy individuals. In this paper, the scope of the study was to investigate strange-face illusions in patients with depression. Depressive subtypes are positioned to differ functionally by differential contributions by serotoninergic, noradrenergic, and dopaminergic neurotransmitter circuits [6]. The altered cognitive and affective processing in depression has been associated with disruption of frontotemporal and frontosubcortical networks [7]. Depression is characterized by maladaptive bottom-up processes that are generally perpetuated by attenuated cognitive control [8, 9]. Therefore, the main hypothesis of the present study was that strange-face apparitions, in response to mirror-gazing, should be different in frequency and intensity in depressed patients with respect to healthy controls.

Human faces convey important messages, such as identity, age, sex, eye gaze, and emotional expression, which are relevant to social communication and interpersonal interaction. In face-to-face interactions between the subject and the other, facial expressions by the other and facial recognition of the other's expressions by the subject are reciprocally intertwined through mimicry and subject-other synchronization [10].

Mirror-gazing at one's own face is similar to an interpersonal encounter by the subject (or the subject's ego) with itself (which is the subject's bodily face that is reflected in the mirror), as if the subject were an other [11, 12]. In the case of mirror-gazing, the subject's facial expressions are reflected in the mirror and then perceived and recognized by the subject itself. This dynamic self-reflection can produce, within the subject, recognition-expression or perception-action loops. Hence, mirror-gazing can involve, within the subject, processes of mimicry, synchronization, emotional connectedness, and so forth, that are all implicated during face-to-face interactions.

In relationship to faces, patients with depression show deficits both in facial recognition of emotions and in facial expression of emotions [13–15]. Emotional-processing biases occur to sad faces presented below the level of conscious awareness in depression [16, 17]. Depression patients show deficits in both voluntary and involuntary facial expression of emotions [13]. By influencing the salience of social stimuli, mood-congruent processing biases may contribute to dysfunction in conscious recognitions, expressions, and social interactions in depression [18].

Therefore, a specific hypothesis, which is based on deficits of facial recognition and facial expression of emotions in depression, is that strange-face apparitions should be strongly reduced in patients with depression compared to healthy controls.

From the clinical viewpoint, it may be noted that no study has previously investigated mirror-gazing in depressed patients. Therefore, a simple, standardized test to trigger a reproducible pattern of strange-face apparitions could help in completing the standard psychopathological assessment of patients with depression.

## 2. Materials and Methods

The study was approved by the hospital ethical committee. The experiment was conducted in accordance with the Declaration of Helsinki (1964). All participants provided written informed consent before entry to the study.

### 2.1. Participants

Our clinical sample consisted of thirteen hospitalized patients in “Villa Santa Chiara” Clinic in Verona, Italy. They were 5 men and 8 women (mean age 50.0 years; SD 14.2) with depression. Clinicians who have many years of practice with psychiatric patients did their diagnoses according to DSM-IV-R criteria (American Medical Association, 2004). Controls were 13 individuals recruited from hospital workers. They were 5 men and 8 women (mean age 40.2 years; SD 13.0) who declared no history of neurological or psychiatric impairment. Depressed and control individuals were volunteers; they were naïve to the research aim.

### 2.2. Procedure

In the experiment, participants were tested in random order. The experimenter was blind about the condition of the participant either a patient or a control individual.

### 2.3. Mirror-Gazing Test (MGT)

MGT was conducted in a darkened room, 5 m × 5 m. The walls of the room were painted light gray. A mirror (0.5 m × 0.5 m) was mounted on a tripod and placed in the center of the room. The subjects were seated at a distance of 0.4 m in front of the mirror. The room was lit only by a halogen light bulb (12 V, 20 W). The bulb was mounted on a spotlight placed 1.2 m behind the subjects so that they could not see it. The light bulb beam was directed toward the floor (about a distance of 5 cm from bulb to floor), in order to avoid direct lighting. This indirect illumination provided diffuse lighting over the whole room. The face was lit relatively uniformly at about 0.2 cd m^−2^ (digital photometer Pantec LM-20 by Carlo Gavazzi, Milano, Italy). All the fine facial features could be perceived in detail; colors were attenuated.

With the subject seated in front of the mirror, the experimenter explained the task: “*Your task is look at your face in the mirror. You should keep staring into your eyes. The task will last seven minutes.*” During the MGT, the subjects' perceptions were qualitatively and quantitatively assessed. The number and latency of abnormal perceptions were evaluated by recording event-related responses to apparitional experiences. Every time subjects had an abnormal perception, they had to press a button and their responses were recorded and digitally stored. The experimenter told participants how to use the button using the following words: “*During the seven minutes while you are looking at your face in the mirror and staring at your eyes you may or may not notice changes in your face. If you notice a change then press the button and hold it down for as long as the change lasts. If you do not notice any changes then do not press the button.*” Subjects were then asked if they understood the task, and, after the experimenter had further clarified and explained unclear points, the task began. The mirror-gazing session lasted seven minutes.

When the 7-minute MGT ended, the experimenter assessed patients and controls with a specifically designed questionnaireand interviewed them asking them to describe abnormal perceptions. The interview comprised the following question: “*What did you see?*” For both patients and controls, the experimenter transcribed the answers.

Lastly, after the interview, the participants answered four five-point Likert-type scale sentences: “*How often did you notice anything strange?*”, “*How often did it influence you emotionally?*”, “*How often did it seem real?*”, and “*How often did you see another person in the mirror?*”. Responses given were scored on a five-point Likert-type scale, ranging from “never” (= 0), “rarely” (= 1), to “very often” (= 4). The experimenter transcribed patients' and controls' answers to the questionnaire.

### 2.4. Statistical Analyses

The two groups (patients versus controls) were matched for gender. Instead, the age of participants was not adequately matched between groups, which were different in mean age (*t* = 1.8; *P* = 0.08). Therefore, possible effects of age differences between groups were investigated by including age as covariate variable in statistical analyses.

For event-related responses, the mean onset of the first apparition was defined as the first time the subject pressed the button. The frequency of event-related responses was defined as the number of times subjects pressed the response button, averaged per minute. The mean duration was the mean time they held the response button down. The cumulative duration of apparitions was defined as the sum of durations of apparitions in MGT, averaged per minute (the cumulative duration equals the algebraic product of frequency and mean duration).

The phenomenological descriptions were classified for content into strange-face categories [1]: deformed traits, relatives, unknown persons, archetypal faces, animal faces, and monstrous faces. The number of strange faces was calculated for each subject by counting the number of different types of strange faces described.

Between-subjects ANOVAs were run with a two-level factor (patients versus controls). The effect of age differences between patients and controls was analyzed by inserting age as covariate variable in ANOVAs. All data are expressed as means ± SEM.

## 3. Results and Discussion

### 3.1. Event-Related Responses

Five out of 13 patients (38%) perceived strange-face apparitions; 13 out of 13 healthy controls (100%) perceived strange-face apparitions.

The mean onset of the first apparition (patients 68 ± 23 s versus controls 175 ± 38 s) did not differ significantly between patients and controls. The mean frequency of event-related responses (patients 0.3 ± 0.2 versus controls 0.8 ± 0.2) did not differ significantly between groups. The mean duration of apparitions (patients 1.2 ± 0.4 s versus controls 6.1 ± 1.0 s) was lower in patients than in controls (*F*(1, 23) = 13.9; *P* < 0.001). Age differences between groups had statistically nonsignificant effects.

The trade-off between frequency and duration of subject's responses was verified using the mean cumulative duration of apparitions per minute of MGT that differed significantly between groups (*F*(1, 23) = 4.1; *P* = 0.05). The mean cumulative duration of apparitions was shorter in patients than in controls (patients 1.0 ± 0.6 s min^−1^ versus controls 5.0 ± 1.4 s min^−1^). Age differences between groups had statistically nonsignificant effects.

**Figure 1:**
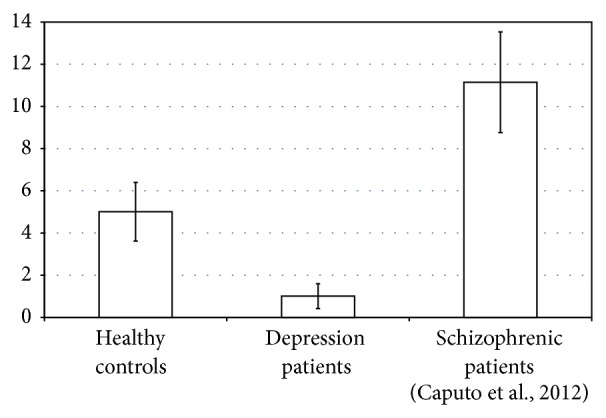
Cumulative duration of apparitions per minute of MGT [s min^−1^].

Results of event-related responses take on more impact when compared with data from schizophrenic patients [5], as shown in Figure 1 (the three groups were actually studied in the same sessions and all participants were tested in blind design).

### 3.2. Phenomenological (Qualitative) Descriptions

During the 7-minute MGT, patients perceived a lower number of strange faces than controls (patients 0.5 ± 0.3 versus controls 1.6 ± 0.3) and this difference was statistically significant (*F*(1, 23) = 4.4; *P* = 0.047). Age differences between groups had statistically nonsignificant effects.

### 3.3. Likert-Type Scale Questionnaire

The sentence “*How often did you notice anything strange?*” was rated to be lower in strength of apparitions by patients than controls (Likert-type scale score of patients 0.5 ± 0.3 versus controls 1.6 ± 0.2). The difference between groups was statistically significant (*F*(1, 23) = 6.6; *P* = 0.017). Age differences between groups had a statistically significant effect (*F*(1, 23) = 6.2; *P* = 0.02).

The sentence “*How often did it influence you emotionally?*” was rated as lower by patients than controls (patients 0.3 ± 0.2 versus controls 1.3 ± 0.2). The difference between groups was statistically significant (*F*(1, 23) = 8.4; *P* = 0.008). Age differences between groups had statistically nonsignificant effects.

The sentence “*How often did it seem real?*” did not differ between patients and controls (patients 0.4 ± 0.2 versus controls 0.7 ± 0.3). The sentence “*How often did you see another person in the mirror?*” did not differ between patients and controls (patients 0.2 ± 0.2 versus controls 0.7 ± 0.3). Age differences between groups had statistically nonsignificant effects.

## 4. Conclusions

Our study provides first evidence showing that mirror-gazing, at a low illumination level, produces less frequent strange-face apparitions in depressed patients than in healthy individuals. Moreover, apparitions were usually of lower intensity and shorter duration in depressed patients than in healthy controls. The hypothesis of the present study is therefore supported by the decreased frequency and duration of event-related responses, decreased number of strange faces, and lower self-evaluation ratings of apparition strength and emotions among the patients. Instead, the age of participants did not influence strange-face apparitions.

The experimental finding that depression patients reported lower ratings of the emotional content of strange-face apparitions than healthy controls can be explained by the general dampening of emotions in depression [7, 8, 19].

The experimental finding that patients reported fewer and less frequent strange-face apparitions than controls can be explained by deficits in emotional facial recognition and emotional facial expression and by deficit in interpersonal interactions of patients with depression [13–15, 18].

Our phenomenological observation of typical depressed patients' behaviour in front of the mirror gives the compelling impression that patients saw their own reflected faces similar to inanimate materials. This behaviour in depression is opposite to intense strange-face hallucinations that can be observed in schizophrenia [5]. In fact, depression patients during mirror-gazing can be described as completely immobile similar to statues of death [20].
